# Web-based training and certification of clinical staff during the randomised clinical trial SafeBoosC-III

**DOI:** 10.1186/s13063-024-08530-x

**Published:** 2024-10-23

**Authors:** Marie Isabel Skov Rasmussen, Mathias Lühr Hansen, Colin Peters, Gorm Greisen, SafeBoosC-III Trial Group, Adelina Pellicer, Afif El-Kuffash, Agata Bargiel, Ana Alarcon, Andrew Hopper, Anita Truttmann, Anja Hergenhan, Anja Klamer, Anna Curley, Anne Marie Heuchan, Anne Smits, Asli Cinar Memisoglu, Barbara Krolak-Olejnik, Beata Rzepecka, Begona Loureiro Gonzales, Beril Yasa, Berndt Urlesberger, Catalina Morales-Betancourt, Chantal Lecart, Christian Gluud, Claudia Knöepfli, Cornelia Hagmann, David Healy, Ebru Ergenekon, Eleftheria Hatzidaki, Elena Bergon-Sendin, Eleni Skylogianni, Elzbieta Rafinska-Wazny, Emmanuele Mastretta, Eugene Dempsey, Eva Valverde, Evangelina Papathoma, Fabio Mosca, Gabriel Dimitriou, Gerhard Pichler, Giovanni Vento, Gitte Holst Hahn, Gunnar Naulaers, Guoqiang Cheng, Hans Fuchs, Hilal Ozkan, Isabel De Las Cuevas, Itziar Serrano-Vinuales, Iwona Sadowska-Krawczenko, Jachym Kucera, Jakub Tkaczyk, Jan Miletin, Jan Sirc, Janus Christian Jakobsen, Jana Baumgartner, Jonathan Mintzer, Julie De Buyst, Karen McCall, Konstantina Tsoni, Kosmas Sarafidis, Lars Bender, Laura Serrano Lopez, Le Wang, Liesbeth Thewissen, Lin Huijia, Lina Chalak, Ling Yang, Luc Cornette, Luis Arruza, Maria Wilinska, Mariana Baserga, Marta Mencia Ybarra, Marta Teresa Palacio, Martin Stocker, Massimo Agosti, Merih Cetinkaya, Miguel Alsina, Monica Fumagalli, Munaf M. Kadri, Mustafa Senol Akin, Münevver Baş, Nilgun Koksal, Olalla Otero Vaccarello, Olivier Baud, Pamela Zafra, Peter Agergaard, Peter Korcek, Pierre Maton, Rebeca Sanchez-Salmador, Ruth del Rio Florentino, Ryszard Lauterbach, Salvador Piris Borregas, Saudamini Nesargi, Serife Suna, Shashidhar Appaji Rao, Shujuan Zeng, Silvia Pisoni, Simon Hyttel-Sørensen, Sinem Gulcan Kersin, Siv Fredly, Suna Oguz, Sylwia Marciniak, Tanja Karen, Tomasz Szczapa, Tone Nordvik, Veronika Karadyova, Xiaoyan Gao, Xin Xu, Zachary Vesoulis, Zhang Peng, Zhaoqing Yin

**Affiliations:** 1 Department of Neonatology, Copenhagen University Hospital - Rigshospitalet, Copenhagen, Denmark; 2 Copenhagen Trial Unit, Centre for Clinical Intervention Research, The Capital Region, Copenhagen University Hospital – Rigshospitalet, Copenhagen, Denmark; 3 Department of Neonatology, Royal Hospital for Children, Glasgow, UK

## Abstract

**Background:**

SafeBoosC-III is a pragmatic, multinational clinical trial evaluating cerebral oximetry-guided treatment for extremely preterm infants. In total, 1601 infants were randomised across 70 sites in Asia, Europe, and USA. To enhance data quality and patient care, a web-based training program was implemented for staff. We now report on the processes.

**Methods:**

All training modules consisted of initial learning material followed by a case-based quiz, with elaborate responses to correct as well as to wrong answers. Modules covered trial introduction, cerebral oximetry monitoring, treatment guidelines, cerebral ultrasound, and Good Clinical Practice. The introduction module was accessible in eight languages on an online platform, while language versions varied for other modules, due to different needs. Certification was earned upon module completion, relevant to the staff category. The training was not mandatory, but for motivational purposes, principal investigators continuously received local certification rate reports.

**Results:**

A total of 926 out of 2347 staff (39%) obtained certification. Amongst 295 staff who completed the evaluation, 83% rated the program as overall good and 94% found it relevant to clinical practice. Sites exhibited varying certification rates, with 10 at 0%, 43 between 0.1 and 79.9%, and 17 exceeding 80%. There was no correlation between the rate of certification in individual sites and how often the clinical management was changed due to cerebral hypoxia nor a correlation to site-specific estimates of the intervention effect.

**Conclusion:**

Despite language barriers and a low budget, our web-based training and certification program proved feasible. Only a minority of sites reached 80% certification of staff and an impact on the trial could not be detected.

**Trial registration:**

The SafeBoosC-III trial is registered at ClinicalTrials.gov NCT03770741. The first participant was randomised in June 2019 and recruitment was completed in December 2021.

**Supplementary Information:**

The online version contains supplementary material available at 10.1186/s13063-024-08530-x.

## What did we know?

- Training clinical staff is important to ensure high quality of clinical trials
- Complex interventions represent a special challenge
- Web-based training may be a pragmatic solution

## What did this study contribute?

- The web-based training program proved feasible in an international, multicentre study with a low budget
- The certification rate varied substantially between sites, but the value of the program was appreciated by those who gave feedback
- There was no correlation between the certification rate at individual sites and how often a change of clinical management due to cerebral hypoxia was reported nor a correlation with the site-specific estimate of intervention effect

## Background

Conducting multicenter randomised clinical trials (RCTs) is a challenge which requires a high level of coordination and training. For RCTs evaluating the effect of a complex intervention, training of staff is essential to ensure that the trial interventions are implemented consistently and with high quality. Although it is stated in the international Good Clinical Practice (GCP) guidelines that staff involved in research “should be qualified by education, training and experience to perform his or her respective task(s)” the processes of delivering training are rarely reported. Relevant studies focus mainly on inter-rater reliability [1–4]. To our knowledge, only few have reported on the process of implementing an intervention training program [5, 6].

The multi-centre, international, pragmatic SafeBoosC-III trial tested if treatment guided by cerebral oximetry monitoring with near-infrared spectroscopy (NIRS) during the first 72 h after birth could decrease the risk of death or severe brain injury at 36 weeks’ post menstrual age in extremely preterm infants. A total of 1601 infants were randomised across 70 sites from Europe, USA, China and India. No significant differences in outcomes were found between the cerebral oximetry and usual care group [7].

The trial evaluated the effect of a complex intervention (monitoring of cerebral oxygenation and responding appropriately and context-dependent on cerebral hypoxia during the first 72 h after birth), and used a primary outcome that involved doing and reading repeated dynamic ultrasound scanning of the brain until 36 weeks of post-menstrual age. Thus, training of clinical staff involved in the care of trial participants as well as outcome assessment was important. We expected differences in demographics, language, and staff training to be a challenge however, the limited funding of the SafeBoosC-III trial was a specific challenge. Previous experience with the intervention in the 70 hospitals varied greatly.

We planned to overcome this by offering a web-based training and certification program to all clinical staff involved in the care of the trial participants. A web-based approach has several advantages over traditional classroom training including increased accessibility, standardisation, cost-effectiveness, and scalability. As the SafeBoosC-III trial was a pragmatic trial, we set out to create a web-based training program which would reflect a real-world setting. The aim was to train staff sufficiently with minimal time investment from their local research teams and to maximise participation.

Before the initiation of the SafeBoosC-III trial, the web-based training module on cerebral oximetry monitoring was piloted by 81 doctors and nurses across China, Spain and Denmark. A description of this process has been published previously [6]. The results of this piloting revealed discrepancies between learning material and quiz questions, lack of clarity, and technical issues. All training modules were revised based on this. We now report on the production and use of the final, large-scale, multi-language web-based training program. Furthermore, recommendations for training and evaluating clinical staff in large multi-centre studies are provided.

## Methods

All modules were designed as integrated training and certification modules, with each module consisting of learning material and a quiz. The quizzes were built over an adaptive framework to recognise prior learning, as correct answers would take users through the modules faster. Detailed feedback was provided on incorrect as well as correct answers thereby delivering both formative assessment and summative assessment within the quiz. The teaching methodology was case-based, and the quiz asked questions that could appear during the clinical care of infants enrolled in the trial. The full description of the development of the modules will not be further outlined in this paper as this process has been published previously [6].

### Modules

The training program consisted of 5 modules: (1) The introduction module gave a brief description of the phase II trial, the rationale behind the present SafeBoosC-III trial and methodology; (2) the NIRS module introduced important aspects of cerebral oximetry in preterm babies with both physiology as well as practical information on how to handle the NIRS sensor; (3) the Treatment Guideline module introduced the pathophysiology of cerebral hypoxia, relevant clinical assessment as well as interventions to restore normal cerebral oxygenation; (4) the Cerebral Ultrasound module introduced relevant aspects of cerebral sonography, being the primary outcome assessment in the trial; (5) the GCP module including central parts of the local monitoring plan of the trial.

To obtain certification, nurses had to complete the Introduction as well as the NIRS module. Neonatologists had to complete all modules except for the module on GCP; this was only required to be completed by the principal investigators. Radiologists were required to complete the module on Cerebral Ultrasound. In the fall of 2020, it was changed so the Cerebral Ultrasound module was no longer required for neonatologists, since many sites reported that ultrasound assessments were mainly carried out by radiologists. The training modules were designed to ensure that completion would provide sufficient qualification, allowing principal investigators to be confident that staff were adequately prepared to carry out their roles according to ICH-GCP standards.

### Virtual learning environment

The modules were hosted in Moodle, a virtual learning environment (Moodle Pty Ltd, West Perth, WA, Australia). This shareware software has been used for online medical training previously [8, 9]. Principal investigators were sent instructions and a specific password to enter the program and were thereafter in charge of distributing this to relevant staff. Users registered in Moodle through a provided URL. During registration, users were asked to decide on their first language, place of work and clinical position, and consented to data from the training and certification process being anonymously logged and used for analysis and publication. It was possible to revisit the site as often as needed to complete the modules. Once completing all the modules relevant to their clinical position, users were asked to report their experience with the modules. The questionnaire used for this report was only available in English.

The modules were built in the software Articulate Storyline (Articulate, New York, NY, US). Expenses to Copenhagen University Hospital e-learning section were 17.000 Euro and covered consultancy on pedagogic methodology as well as programming. This process led to the English modules. External consultants were hired to implement translations into the software versions. The platform Moodle cost an annual subscription of approximately 2700 Euro to host the training program.

### Translation

As SafeBoosC-III was a multinational trial, language barriers presented a significant challenge. National coordinators were in charge of translations of modules. They also decided what modules should be translated, and therefore not all modules were translated. In most countries, the doctors completed the English versions of the Cerebral Ultrasound module as well as the Treatment Guideline module while the Introduction and NIRS module were translated into local languages. The translation process was done in a manual and simple manner by national coordinators. Given limited resources, the quality of the translations was not evaluated by external linguistic experts nor were checked by back-translations. The translations as well as the English template module with Articulate Storyline programming were then sent to external programmers through the freelance service Fiverr (Fiverr, Tel Aviv, Israel). The cost was approximately 100 Euro per module.

### Users

All staff (nurses, neonatologist, radiologist and principal investigators) that were listed on the training and delegation logs at each site by the principal investigator for the purpose of the local GCP monitoring, were invited to participate in the web-based training and certification program. Although participation was highly encouraged, it was not made mandatory, as the trial steering committee feared this would delay the initiation of the trial.

### Monitoring

To continuously monitor certification rates and to motivate local training, a report was sent out to principal investigators before initiation of randomisation, and at 1, 3 and 6 months after initiation of randomisation of the first participant at that site. About 3–4 h per week was spent conducting this monitoring. The principal investigator could request certificates for clinical staff who had completed the training and certification, which could be used for local purposes.

### Data analysis

Quantitative data was analysed using the software SPSS (SPSS Inc. Released 2009. PASW Statistics for Windows, Version 18.0. Chicago: SPSS Inc). Analysis of close-ended answers from the evaluation module was presented using descriptive statistics while open-ended questions were analysed following the principles of thematic analysis as described by Braun and Clarke [10]. A six-step process was followed which entails a systematic approach of recognising and labelling units of meaning using codes, which can be individual words or groups of words that convey a specific meaning. Subsequently, the data was examined to identify patterns, and the information was categorised into overarching themes that encapsulate the principal concepts and their relationships.

### Ethics

The web-based training and certification program was described in the protocol when applying for ethics approval to conduct the SafeBoosC-III trial. When creating a profile, users consented to personal data to be stored (name, workplace, clinical position and email).

## Results

Overall, 926 staff out of the total of 2347 listed on the 70 training and delegation logs obtained certification. There were 1405 registered users on the training website, i.e. 66% of users registered reached certification.

### User evaluation

**Fig. 1:**
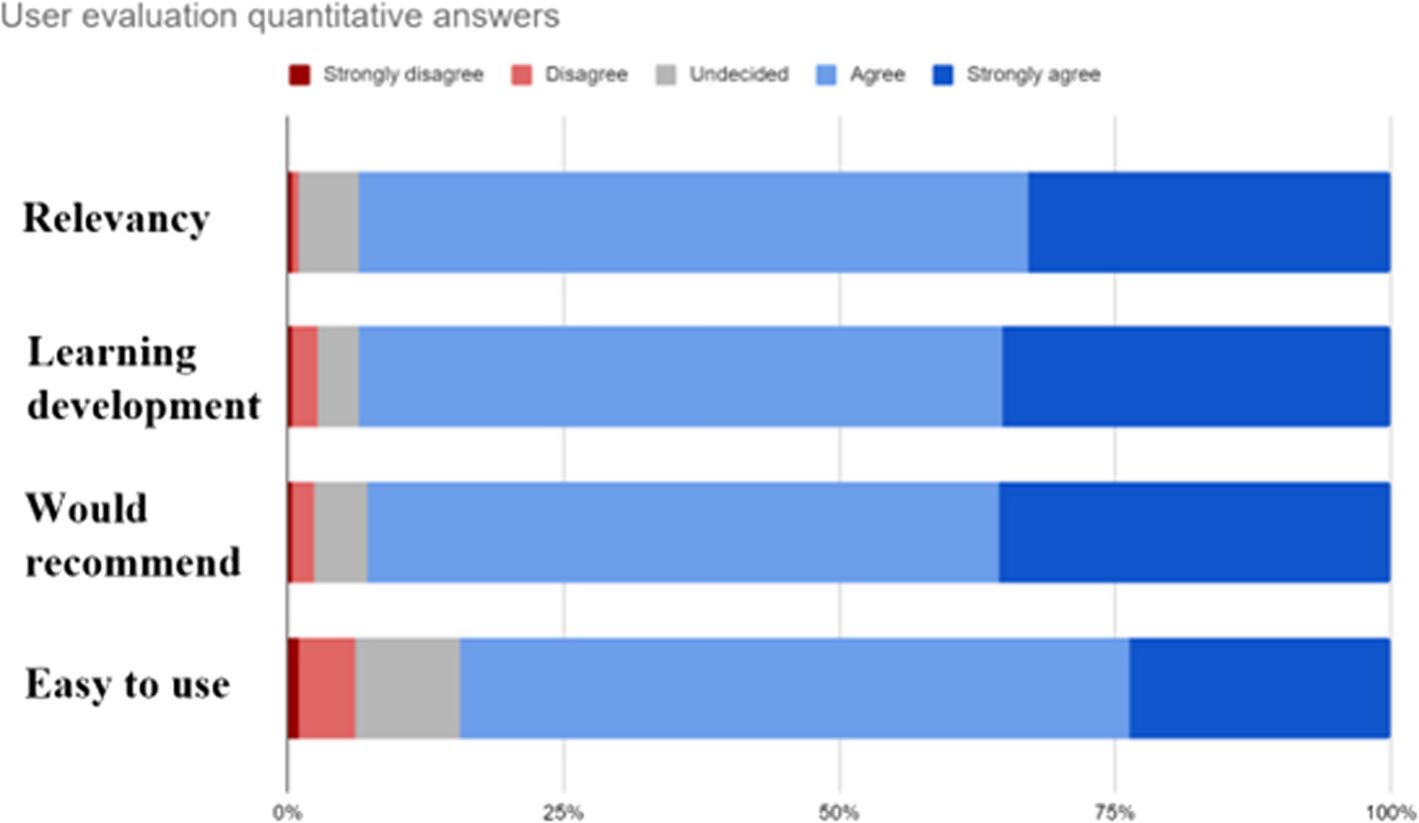
Quantitative answers from the user evaluation (*n* = 295). Users were asked on a Likert scale if they agreed or disagreed with questions (here illustrated as themes), regarding the web-based training and certification

The user evaluation was completed anonymously by 295 users. Overall, 83% rated the overall quality of the online course as either good or very good. Between 92 and 93% agreed or strongly agreed that the course was relevant for clinical practice, that they had learned more about cerebral oxygenation than before and that they would recommend it to colleagues (Fig. 1).

**Fig. 2:**
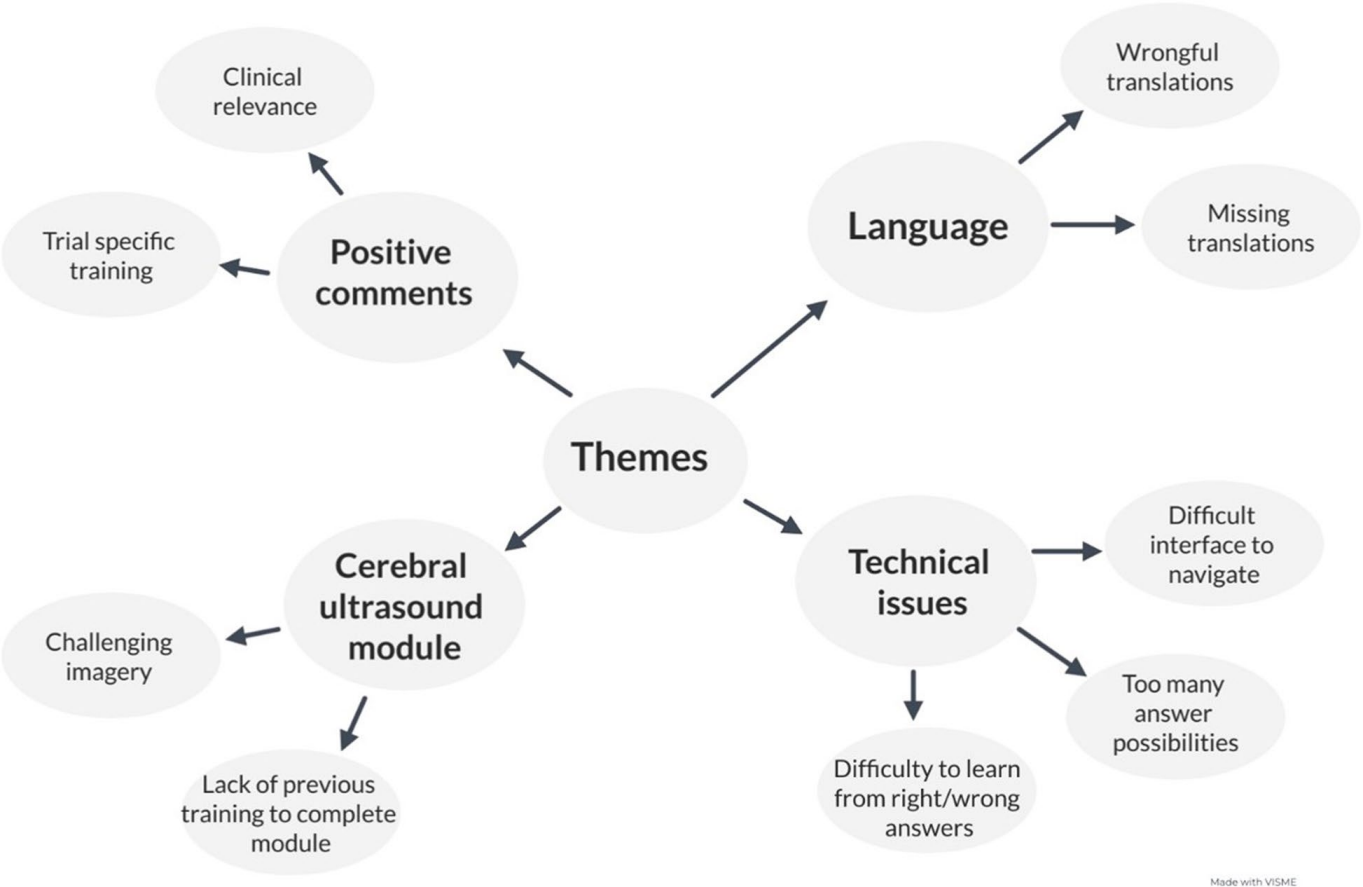
Overview of themes and sub-themes in the thematic analysis

The thematic analysis was derived from 33 open-ended answers from the evaluation module and resulted in four essential themes, accompanied by sub-themes. The four themes were (1) language, (2) technical issues, (3) Cerebral Ultrasound module, and (4) positive comments. An overview of the themes can be found in Fig. 2.

### Language

Three users commented on some difficulties in understanding the language.

“Sometimes the wording is a little difficult to understand.” and “Some questions asked provide incomplete and badly articulated descriptions and answers.”

Not all modules were available in all languages, which frustrated a few users (*n*=3)

“It wasn’t really easy for me because it’s in English, and I speak Polish. Some words were quite difficult for me.”

### Technical issues

The interface and format of the training program were commented on multiple times (*n*=6). Some thought it was hard to navigate within the quiz, for example when trying to learn from wrong answers.

“It’s not possible to go back after getting the wrong answer. It should be good to see both wrong and correct answer.”

A user commented that correct answers should be provided if the user does not get it right after multiple attempts.

Two users complained that the webpage was slow and three users that it was not easy to switch between modules.

“The course grading and progress is extremely hard to navigate. I am unsure of what is done and what still needs to be done.”

### Cerebral ultrasound

Three users commented on the academic level of the cerebral ultrasound module noting that the ultrasound images were too challenging.

“For clinicians, the ultrasound module is a bit difficult, mainly for image recognition.**”**

Five users commented that they did not have much previous training and therefore did not feel they were equipped.

“I found the evaluation of cerebellum difficult. It is not routine in our department which? might influence the final evaluation of cerebral outcome” and “I felt the courses to be very informative but we have not been trained in reading ultrasounds so it was intimidating”

### Positive comments

A total of eight users had positive comments towards to training program and for example applauded the clinical relevancy.

“Very well put together course, based on common clinical findings and situations!”

One user also commented on the trial relevancy of the training program.

“Good and partially tough questions especially in the clinical treatment part. I feel more confident to carry out the study now. Thank you for a very interesting course!”

### Effect on trial

Ten sites had a certification rate of 0%; 43 sites had a certification rate between 0.1 and 79.9%; and 17 sites had a certification rate over 80%. The average number of randomisations in the 0% group was 16; 23 for the 0.1–79.9% group; and 24 for the >80% group. The groups randomised 14%, 62% and 24%, respectively, of all 1601 participants in the trial. The average certification rate across doctors and nurses increased by 14% from before randomisation of the first participant at each site to 6 months after. In general, doctors had a higher certification rate than nurses (Table 1). The sites with a high certification rate amongst doctors also had a higher certification amongst nurses (Kendall’s tau 0.608). In total, 921 users completed the NIRS model, meaning that 40% of staff obtained information regarding intervention (Table 1). English was the most used language followed by Spanish (Table 2).

**Table 1:** Total number of completions for each module

	**Introduction**	**NIRS**	**Treatment guideline**	**Cerebral ultrasound**	**Good Clinical Practice**
**Neonatologist**	50% (401/810)	50% (404/810)	49% (397/810)	46% (372/810)	10% (85/810)
**Nurses**	36% (537/1493)	34% (517/1493)	0.4% (6/1493)	0.4% (6/1493)	0% (0/1493)
**Radiologist**	0.1% (2/44)	0% (0/44)	0% (0/44)	70% (31/44)	0% (0/44)

**Table 2:** Number of completions per language version (NA = language version was not available). Some modules were completed in multiple languages by the same user

	**Introduction**	**NIRS**	**Treatment guideline**	**Cerebral ultrasound**	**Good Clinical Practice**
**English**	751	736	357	390	124
**Spanish**	141	145	53	NA	NA
**Turkish**	16	19	8	NA	NA
**Chinese**	83	86	49	50	14
**Italian**	36	38	NA	NA	NA
**German**	7	13	NA	NA	NA
**French**	89	73	NA	NA	NA
**Czech**	7	NA	NA	NA	NA

**Fig. 3:**
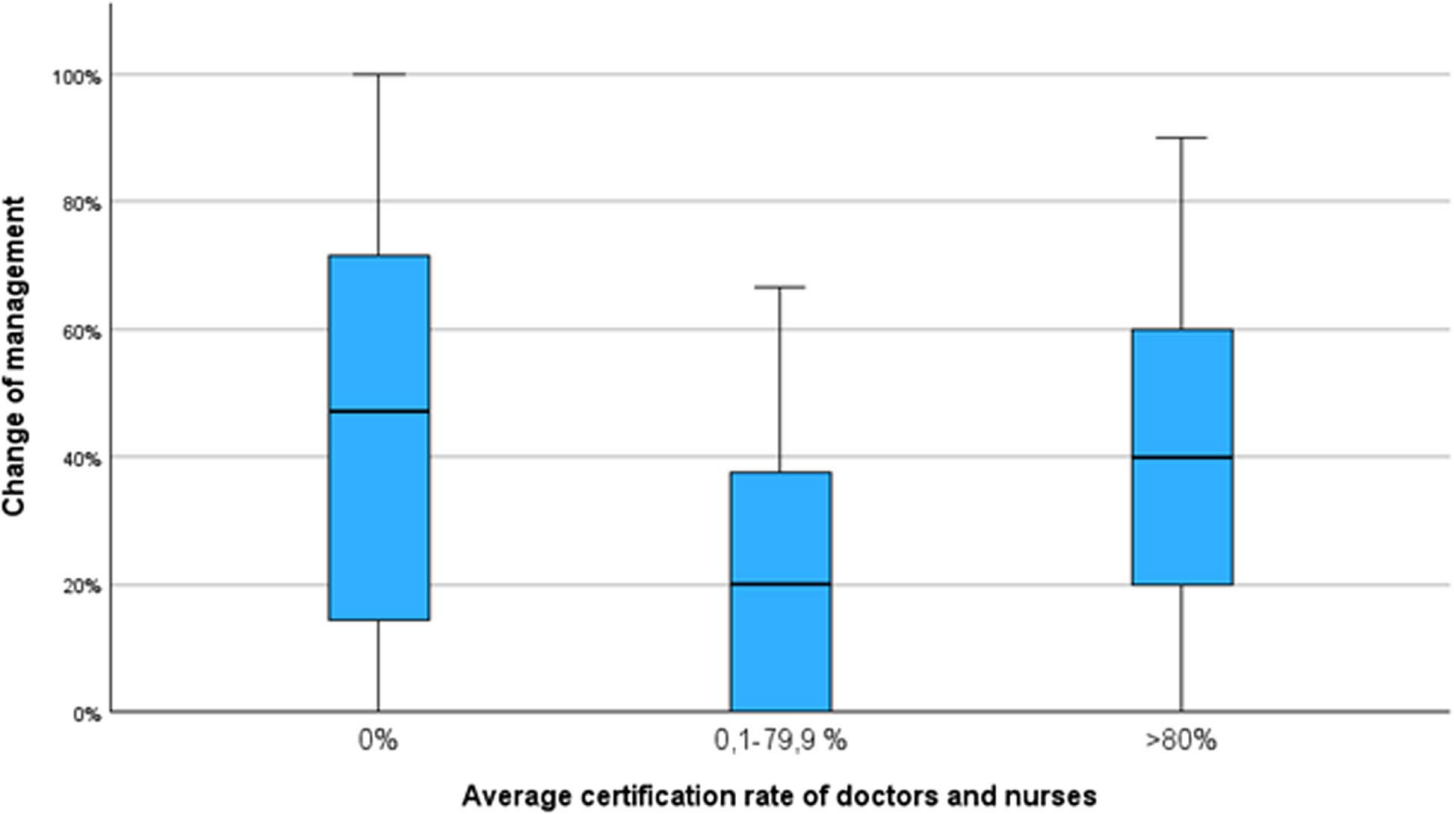
The lack of association between staff certification rates and the site-specific statistics of the proportion of participants in the SafeBoosC-III trial’s experimental group with a report of change of clinical management

**Fig. 4:**
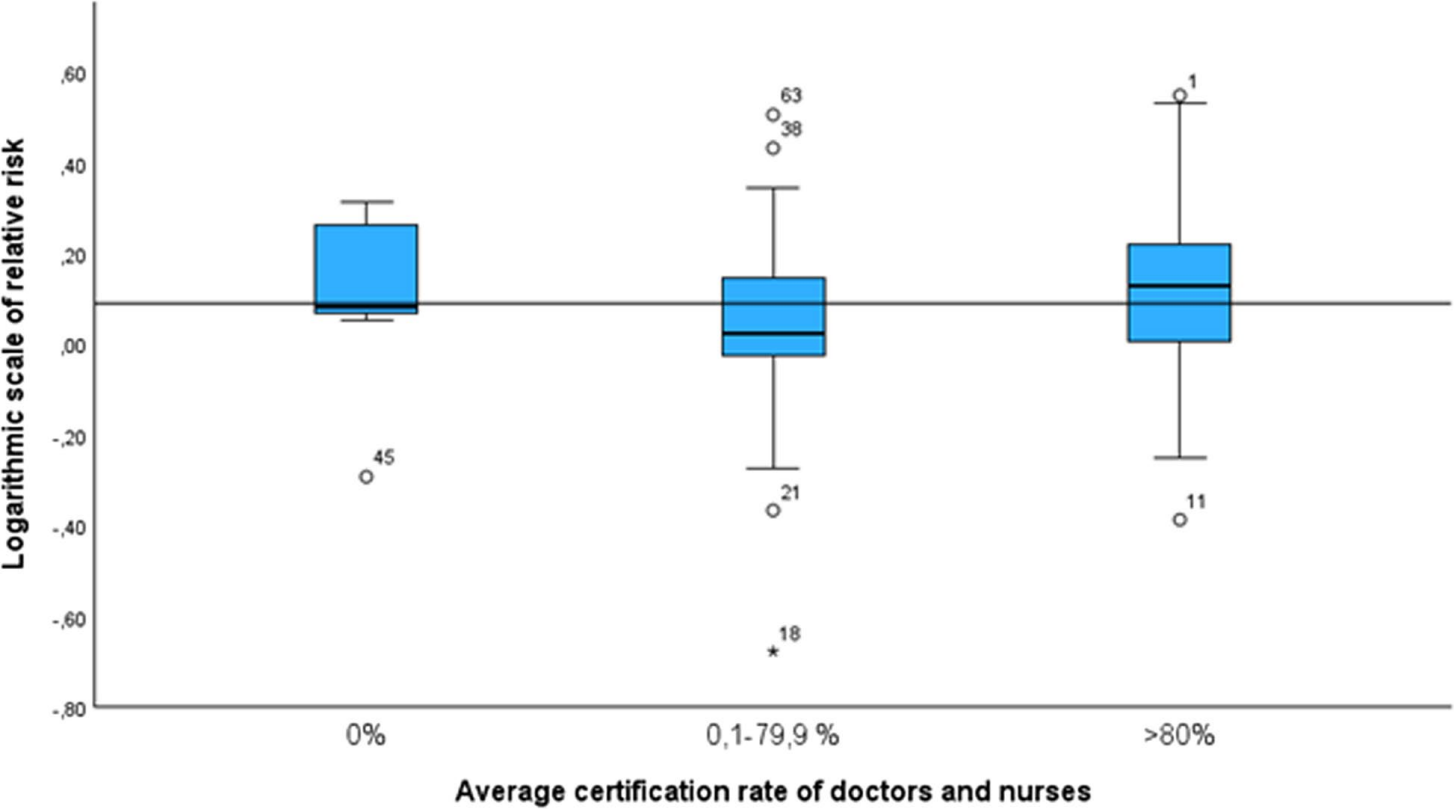
The lack of association between staff certification rates and the site-specific statistics of the intervention effect (relative risk of death or survival with severe brain injury)

There was no significant correlation between the number of randomisations per site and the mean certification rate of nurses and doctors (Kendall’s tau 0.114 and 0.057, respectively). Furthermore, there was no significant correlation between the mean certification rate of nurses and doctors and change in clinical management due to cerebral hypoxia (Kendall’s tau 0.146 and 0.133, respectively) (Fig. 3). Lastly, there was no significant correlation between the site-specific estimate of the effect on the primary outcome of the trial (survival without major cerebral injury) (Kendall’s tau 0.099 and −0.018, respectively) (Fig. 4).

## Discussion

This paper outlines the methods and processes involved in implementing a web-based training and certification program for the SafeBoosC-III trial. Despite encountering challenges such as differences in clinical practices, language barriers, and a limited budget, our training program demonstrated its feasibility in an international trial setting. Users expressed the program's clinical relevance and a high level of satisfaction. Only a minority of sites reached certification of 80% of staff, and an impact on the trial and intervention effect could not be detected. Drawing from our experiences, we offer several recommendations for the training of clinical staff in multicenter studies.

### Pilot and iterative development

In accordance with the PRECIS-2 tool, we aimed at delivering a high-quality intervention and conducting a pragmatic trial that reflected real-world constraints [11]. Given the scarcity of literature documenting this, we adopted a learning by doing approach. We conducted pilot testing, made adjustments based on feedback and executed the program [6]. Recommendation: Prior to full implementation, conduct a pilot study involving diverse staff from different clinical settings. Based on this, refine the training modules to improve clarity and effectiveness. We highly recommend documenting this process for other trialists to learn from.

### Real words scenarios and immediate feedback

We developed integrated modules incorporating learning material and quizzes which proved effective. The immediate feedback on case-based scenarios and high clinical relevance was also appreciated and aligned with previous successful approaches. Recommendations: Integrated modules comprising both learning material and quizzes featuring real-world scenarios with immediate feedback could be a way to increase engagement and participation.

### Enhancing communication with users

Communication with clinical staff was conveyed through principal investigators as the trial centre did not have direct contact with staff. A total of 39% (926/2347) staff, achieved certification, however, 66% (926/1405) of those who created accounts reached certification. This may be the real measure of feasibility in regards to the effectiveness of the training program. Recommendation: Consider prioritising direct communication with clinical staff, perhaps through principal investigators supplying email addresses. Introduce a reminder system to encourage regular engagement and ensure all staff receive and comprehend training instructions. Integrating more interactive elements or peer-learning opportunities into the training program could enhance motivation and participation amongst clinical staff.

### User-friendly interface

Concerns were raised regarding the web interface, with feedback highlighting the slow performance of the platform and difficulty in navigating between modules. We could have built a more technically robust platform, had we used IT professionals for this task or done user acceptability testing to ensure easy navigation. Recommendation: Develop and prioritise a user-friendly and intuitive interface, allowing users to easily navigate through modules, track their progress, and revisit modules as needed.

### Customised content for staff categories

Acknowledging variations in staff roles and prior knowledge, we tailored our modules to meet different learning needs. The thematic analysis indicated that some doctors found the cerebral ultrasound module challenging due to limited prior training, leading to feelings of unease. The limited availability of modules in all languages may have influenced certification rates, and a correlation between certification rates and language accessibility may be possible; however, we lack data to investigate this further. Differences in practice across countries, where either neonatologists or radiologists perform these scans, prompted our decision to exclude it as a requirement for neonatologists. In total, however, 59 out of 70 sites had at least one doctor (either neonatologist or radiologist) complete the cerebral ultrasound module. Recommendation: Tailor the content of the training program to the specific staff roles at different sites. Ensure that each module meets the learning needs of the respective group according to their tasks involved in the care of trial participants. Explore language availability needs.

### Consider offering alternative training possibilities and extended monitoring of such

Initially, the question of making training and subsequent certification mandatory was discussed, but we refrained from this due to concerns on decreasing trial feasibility. SafeBoosC-III was a pragmatic trial with a small budget. The principal investigator or national coordinator had to raise funding for all local costs [7]. When running a trial on these terms, it is also important to minimise the workload for investigators and clinical staff. Requiring all relevant staff to complete web-based training and certification before recruitment would have significantly increased the workload, especially for principal investigators, who would need to oversee and motivate staff. This added burden could have delayed trial preparations and reduced feasibility, potentially causing some sites to withdraw or decline participation.

Although less than 50% of staff obtained web-based certification, this does not fully capture the level of training during trial preparations. Some sites conducted group online training or used quiz materials for classroom sessions, and other alternative training methods may have been used. Monitoring these methods could improve training reporting. Notably, 10 sites had a 0% certification rate, but we did not collect data on alternative training approaches.

Recommendation: Depending on the trial’s specific context, consider customising training material and provide alternative ways to educate and train staff, in order to fulfil the needs of individual sites. Consider monitoring the use of the different education and training modalities, to obtain better reporting on the actual education and training. A solution could be to have staff sign off on the training and delegation log, if they have done online, classroom, clinical training, etc.

### Data collection

We did not collect data from within the modules, such as time to completion, attempts, questions used etc. This restricted our ability to analyse user learning behaviours comprehensively. Recommendation: To enhance the effectiveness of similar training programs in the future, consider collecting more comprehensive quantitative data, such as pre- and post-training knowledge assessments, module-specific completion rates, and user demographics.

### A discussion of the certification rates and the neutral result of the SafeBoosC-III trial

The effect of the SafeBoosC-III trial intervention on the primary outcome was neutral. The pragmatic nature of the trial may also set the scene for the training program. We wanted to test how the intervention would perform when implemented in a real-world setting, managed by staff with various experiences in different clinical settings. We aimed for a generalizable result. Only 17 out of 70 sites had an average certification rate exceeding 80%. We suggest that this gives a realistic image of the level of training that may be obtained for clinical implementation of cerebral oximetry—involving varying degrees of participation and engagement. It is possible that the SafeBoosC-III trial did not realise the potential for reduction of cerebral hypoxia shown in the SafeBoosC-II trial, where only eight sites were involved [7, 12]. The correlation analysis, however, did not suggest that higher certification rates translated into more active management of cerebral oxygenation by changes in clinical management of the children in the experimental group, nor into a higher chance of survival without major brain injury. It is possible that the training program contributed to an overall increase in awareness and preparedness amongst staff, but not anything detectable on effect estimates.

Despite language barriers and a low budget, our web-based training and certification program proved feasible and was well-received, however, only a minority of sites reached 80% certification of staff, and an impact on the trial could not be detected. Overall, we believe that our experiences may provide insights and inspire future trialists.

## Trial status

The SafeBoosC-III trial is registered at ClinicalTrials.gov NCT03770741. The first participant was randomised in June 2019 and recruitment was completed in December 2021.

## Supplementary Information

Supplementary Material 1.

## Data Availability

Not applicable.

## Abbreviations

SafeBoosC-III: Safeguarding the Brain of our Smallest Children phase III
NIRS: Near-infrared spectroscopy
NICU: Neonatal intensive care unit
